# Learned helplessness and learned controllability: from neurobiology to cognitive, emotional and behavioral neurosciences

**DOI:** 10.3389/fpsyt.2025.1600165

**Published:** 2025-08-26

**Authors:** Gustavo E. Tafet, Tomas Ortiz Alonso

**Affiliations:** ^1^ Department of Psychiatry and Behavioral Sciences, Texas A&M University, College Station, TX, United States; ^2^ Universidad Complutense de Madrid, Madrid, Spain

**Keywords:** stress, depression, trauma, resilience, helplessness, controllability

## Abstract

The sustained and prolonged exposure to environmental stressors may provoke different reactions, depending on the subjective perception of control. Repeated perception of lack of control may lead to learned helplessness, which has been linked to the development of depression. It has been shown that learned helplessness is associated with increased activation of the serotonergic dorsal raphe nucleus (DRN) and the amygdala, as well as the decreased activation in certain regions of the prefrontal cortex (PFC), such as the dorsolateral (DL-PFC) and the ventromedial prefrontal cortex (VM-PFC). Perception of control has been associated with increased activation of the DL-PFC and the VM-PFC, decreased activation of the DRN and the amygdala, as well as increased activation of the serotonergic medial raphe nucleus (MRN). It is also associated with decreased activation of the ventral hippocampus and increased activation of the ventral striatum, including the nucleus accumbens (NAc). Functionally, perception of control promotes the implementation of active coping strategies, characterized by efficient cognitive-emotional processing, effective decision making, and goal directed actions, that in turn may lead to lower anxiety, greater tolerance of adverse events, emotional stability, and increased resilience. Just as it has been demonstrated that helplessness may result from a learning process, it could be hypothesized that controllability may also be trained and learned. If so, learned controllability could be taught to enhance resilience and provide invaluable resources ultimately reducing depressive symptoms and improving overall well-being. By fostering a sense of controllability, where individuals learn to associate their actions with desired outcomes, it may be possible to counteract the consequences of “learned helplessness” aspects of depression. This could provide direction in development of novel interventions aimed at promoting skill acquisition, problem-solving strategies, and adaptive decision-making, thereby restoring a sense of agency and resilience in the face of adversity.

## Introduction

Stress represents a normal aspect of life, where perceived environmental stimuli, known as stressors, provoke an array of adaptive responses aimed at protecting or restoring psycho-physiological equilibrium of the organism. The role of stress in the origin and the development of clinical conditions, such as depression and anxiety disorders, has been extensively investigated (1–4), focusing on both psychological and neurobiological processes involved. In this context, the long-lasting effects of early life trauma, and detrimental effects of sustained and prolonged environmental stressors, have been universally acknowledged (4–7). Thus, environmental stressors can trigger different responses, which depend on the concerted activation of diverse neural structures and circuits that support both adaptive and maladaptive physiological and psychological processes.

It has been shown that sustained and prolonged exposure to uncontrollable stressors in rodents can lead to the development of “helplessness”. Since these changes in animal behavior were learned, through exposure to repeated uncontrollable stressful stimuli, it was termed “learned helplessness”, a cognitive/emotional response suggesting a loss of ability to exert behavioral control over environmental stressors, even when this control may be possible. (8). It has been suggested that this may lead to decreased motivation to exert any effort to cope with perceived stressors, which has been hypothesized, in turn, to lead to the development of depression in humans (9). In contrast, animal studies have shown that exerting control in response to stressful situations can lead to the development of adaptive and effective behavioral responses (10, 11). Moreover, human studies suggest that perception of control contributes to the development of effective cognitive and emotional processing, leading to the development of more effective coping strategies, and goal-oriented behavioral responses (12–14). In this regard, subjective perception of control may depend on different factors, some of them associated with characteristics of environmental stressors, others related to the available resources to cope with them, and some on individual characteristics, like temperament. In this regard, previous experiences associated with successful responses, based on effective coping strategies and positive outcomes, may provide elements to shape a subjective perception of control, which may lead to the development of “learned controllability”. If so, learned controllability could be potentially taught to enhance resilience and provide invaluable resources as a potential treatment for learned helplessness, counteracting its cognitive and emotional consequences, ultimately reducing depressive symptoms and improving overall well-being. By fostering a sense of controllability, where individuals learn to associate their actions with desired outcomes, it may be possible to counteract the consequences of “learned helplessness” aspects of depression. This approach could provide novel directions in developing interventions aimed at promoting skill acquisition, problem-solving strategies, and more adaptive decision-making, thereby restoring the sense of agency and resilience in the face of adversity. Moreover, reinforcing controllability may provide individuals with the psychological resilience needed to cope with future stressors, thereby preventing relapses and promoting long-term recovery. In summary, understanding the neurobiological mechanisms and psychological processes implicated in the origin and the development of learned helplessness, and of learned controllability, may provide important information for the development of innovative approaches for the treatment and prevention of depression and anxiety disorders.

## Physiological responses to stress

Early response to acute environmental stressors involves activation of corticotrophin-releasing factor (CRF, also known as corticotrophin-releasing hormone or CRH) neurons in the amygdala, which participate in fear and anger reactions, as well as activation of noradrenergic neurons in the locus coeruleus (LC), which participate in arousal regulation and enhancement of cognitive functions, that further assists in the implementation of adaptive responses (15). This leads to the activation of the autonomic nervous system (ANS) and the subsequent stimulation of the hypothalamic-pituitary-adrenal (HPA) system, both regulated by dedicated neural structures within the central nervous system (CNS) (16, 17). Thus, stimulatory projections from the amygdala, mainly from its central nucleus (CNA), activate the LC, which is a key noradrenergic nucleus reciprocally connected with the amygdala (18), the nucleus of the solitary tract (NTS), the paraventricular nucleus (PVN) of the hypothalamus, and the bed nucleus of the stria terminalis (BNST), which in turn also sends stimulatory projections to the PVN.

The hypothalamic PVN further participates in the activation of the ANS also through stimulatory projections to the NTS, as well as playing a critical role in the activation of the HPA. In sum, the early response to environmental threats involves stimulatory projections from the amygdala, the LC, the NTS, and the PVN activating the sympathetic branch of the ANS, followed by compensatory activation of the para-sympathetic system, and further activation of HPA axis.

Activation of the HPA system starts, as described previously, with stimulatory projections from limbic structures, mainly the amygdala, and indirect projections through the BNST, leading to synthesis and release of CRH, which stimulates the synthesis and release of adrenocorticotropic hormone (ACTH) in the anterior pituitary, which is released to systemic circulation to reach the adrenal cortex, where in turn glucocorticoids, such as cortisol, are produced and released (19, 20). The HPA axis is normally regulated by multiple negative feedback loops, which exert an inhibitory effect on the synthesis and release of CRH and ACTH in the hypothalamic PVN and the pituitary respectively. In addition, cortisol also activates glucocorticoid receptors in the hippocampus, which sends direct inhibitory projections to the PVN, and indirectly via the BNST (21). Repeated exposure to environmental stressors, as it occurs during chronic stress, has been associated with increased activation of the HPA system, which may lead to alteration of physiological negative feedback mechanisms. This may be translated into chronic activation of the HPA system, leading to increased levels of cortisol (4, 22). In this regard, evidence supports the association between chronic stress and depression at the molecular level, where hyperactivity of the HPA axis, with the consequent increase in cortisol levels, represents one of the most consistent findings in both conditions (4, 19, 23, 24).

## Psychological responses to stress

Adaptive responses to stressors involve diverse coping strategies, which require an adequate assessment of perceived stimuli, as well as their contextual features, and the consequent appraisal of available resources to cope with them. This also requires certain perception of control and ability to predict outcomes, which in turn is based on diverse learning processes (25). Subjective perception of ability to predict implies the possibility to anticipate the potential impact of environmental stressors, the assessment of available resources to cope with them, the ability to detect their potential signals in advance, and the capacity to learn from these experiences. At the basic level, this learning process may involve Pavlovian or “classical conditioning”, where perceived signals, previously associated with the occurrence of a reinforcer, may lead to a predictive association between both, with the consequent elicitation of anticipatory responses (25). Subjective perception of “control”, or the lack of it, on the other hand implies the ability to learn about potential associations between certain actions and their consequent results. This learning is based on “instrumental conditioning”, where an association is established between an action and an outcome, which becomes a “reinforcer”. Together, “classical conditioning” allows anticipation, or prediction, of the occurrence of potentially relevant events, whereas “instrumental conditioning” allows to exert certain control over these events, providing the bases for the subjective experience of controllability. The learned association between actions and outcomes plays a critical role in the development of behavioral responses aimed at the satisfaction of personal needs or the accomplishment of desired goals. Hence, instrumental learning may depend on two different mechanisms, which involve different neural pathways: a stimulus-response habit, which implies instrumental learning about an action, which constitutes a behavioral response followed by an expected reward, and a goal-directed process, which implies the acquisition of incentive value by the obtained reward.

Successful adaptive behaviors depend on the ability to detect causal associations between actions and outcomes. This allows the organism to distinguish between environmental events that may be directly associated with these behaviors, and those that may happen independently, and may not be associated with any particular action (26). Successful behavioral strategies may lead to the implementation of actions that have been associated with increased probabilities to generate reinforcing outcomes, and avoidance of those that have been associated with decreased probabilities of these outcomes (27). A goal-directed behavior may depend, not only on the value assigned to associations between actions and outcomes, but also on the probability that outcomes will occur in the presence or absence of these actions (28). This causal association constitutes a “contingency”, which represents the difference between the contingent probability to generate an outcome in response to an action, and the non-contingent probability that the expected outcome may occur independently of that action (26, 29). This suggests, that in response to stressful situations, efficient cognitive and emotional processing is important for the development of a subjective perception of control, which in turn may lead to more efficient behavioral choices. Hence, a subjective sense of predictability and controllability may also contribute to downregulation of excessive physiological responses, including those mediated by the ANS and the HPA system, therefore preventing the development of clinical conditions associated with the chronic impact of environmental stressors, and the consequent maladaptive responses.

## Learned helplessness

As stated earlier, after prolonged exposure to uncontrollable stressful situations, followed by repeated unsuccessful attempts to cope with them, an animal may learn that any effort to avoid or escape similar situations will be in vain. As a result, it may develop “helplessness”, which represents a failure to attempt escape or prevention. This perception of lack of control, despite the fact that control might still be possible, coupled with recurrent experiences of being unable to escape or avoid aversive situations, may lead to “learned helplessness” (30). This phenomenon was originally described in experimental settings, where animals exposed to a series of unavoidable electric shocks later developed an inability to escape or avoid new shocks in a different environment, where escape was possible through behavioral responses. Therefore, this learned condition, not observed in naive animals, but induced by the impact of unsuccessful attempts to escape uncontrollable stressful stimuli, came to be known as “learned helplessness” (8). Hence, the perception that efforts to control a stressful situation are ineffective, learned from previous unsuccessful experiences, may bias against objective assessment, suggesting that efforts would be worthwhile. In humans, it implies that individuals who have learned that outcomes are independent of their actions might feel that their efforts are ineffective and irrelevant. It has been postulated that the critical factor here was the lack of control the organism experienced over stressful events, which in turn may lead to learned helplessness (31). Hence, learned helplessness may arise from exposure to uncontrollable stressors, depending on the extent to which these stressors are perceived as uncontrollable (32).

The concept of learned helplessness, initially described in rodents, was later tested also in human studies (33–35). The initial studies reported that exposure to inescapable threat can elicit some of the analogous responses to those seen in animals (34–36) and it has been hypothesized that this mechanism might be contributing to the development of psychopathological conditions (9, 37). However, it was also recognized that adapting the concept of learned helplessness from animals to humans required taking into account unique aspects related to human cognition and attributional styles (37, 38). In humans, helplessness involves causal attributions, which can be perceived as either internal or external, relating to personal or environmental factors. These attributions can be seen as stable or unstable, meaning they might be seen as enduring conditions or factors that could change with experiences. Additionally, attributions can be global or specific, affecting either all aspects of life or only particular areas. (37, 39). In this regard, subsequent research in humans demonstrated that learned helplessness may occur if individuals perceive a lack of contingency between actions and outcomes in a certain context, which not necessarily will apply in a different context (38). In addition, it has been shown that learned helplessness may lead to decreased voluntary response initiation and decreased interest in pleasurable stimuli, or anhedonia, which are both symptoms typically observed in depression.

## Learned controllability

It has been suggested that controllability may be developed through a learning process, which involves information processing about stressful situations, behavioral responses, and the associations between them. These associations may play a critical role in conferring controllability and its protective effect when coping with stressful situations (14, 40). Various preclinical studies have demonstrated different behavioral patterns in response to aversive events, depending on the presumed perception of control (8, 10, 41–44). In this regard, animal studies have shown that this suggests the ability to successfully respond to environmental challenges, exerting desired actions in the environment through behavioral responses (14).

In humans, subjective perception of control involves both cognitive and emotional processes, where individuals develop a “belief” in their ability to successfully respond to stressful situations. An individual might perceive a reward, or “reinforcer”, as contingent upon their own behavior, or it may be attributed to external factors. If an individual perceives a causal relationship between their actions and a desired outcome, this reflects a belief in internal control. Conversely, if the individual believes that outcomes are unrelated to their own actions and are mainly influenced by external factors beyond their control, this indicates a belief in external control (45). In line with these observations, it has been proposed that expectations of control may lead to the belief in personal ability to succeed by influencing various aspects of coping behaviors. This includes initiating behavioral responses, mobilizing necessary resources, and sustaining efforts over time in the face of perceived aversive situations (46). It has been suggested that, in contrast to what is observed in “learned helplessness”, the perception of control arises when individuals recognize that their behavioral responses can improve the likelihood of achieving desired outcomes, such as obtaining rewards or terminating adversities, compared to the likelihood of achieving similar outcomes in the absence of these behaviors (9).

In terms of learning theory and “instrumental learning”, underlying both “learned helplessness and “learned controllability”, this process involves the assessment of potential goals, such as obtaining rewards or avoiding punishments, identifying the necessary actions to achieve these goals, and understanding the associations between them. As a result, goal-directed actions emerge as behavioral responses, based on the assessment of action-outcomes contingencies, characterized by the expectation of achieving desired outcomes through these actions (47). Subjective perception of control in humans requires the ability to detect potential changes in action-outcome contingencies, certain flexibility and sensitivity to perceive these changes, and the capability to integrate this information to continue implementing adaptive behavioral responses to successfully act and interact in the face of changing environments (48, 49). Furthermore, learned ability to exert behavioral control over environmental stressors creates a perception of stressors as controllable, which in turn reduces associated negative effects and moderates the potential negative consequences of exposure to subsequent uncontrollable stressors (50).

## Neurobiological mechanisms

Adaptive responses to stress, including defense behaviors such as fight or flight, depend on the concerted activation of different neural structures, such as the PFC, the amygdala, the BNST, the hypothalamus, and the periaqueductal gray (PAG). These structures are integrated into neural circuits, where they are reciprocally connected, and are also regulated by different neurotransmitter systems, including serotonergic, noradrenergic, and dopaminergic projections. In response to stressful situations, the medial PFC, the amygdala, particularly the CNA, the BNST, and the hypothalamus, project stimulatory input to the PAG, which integrates sensory information and participates in the initiation of “active” responses (51, 52). Concurrently, the amygdala and the BNST promote fear and anxiety, which are typical emotional reactions elicited in response to certain environmental stressors (9, 53), particularly if these are perceived as uncontrollable. It has been shown that prolonged and sustained exposure to uncontrollable stressors may lead to altered functioning of these structures, and these alterations have been linked to the development of learned helplessness. In this regard, research has demonstrated that learned helplessness can result in various behavioral consequences, including impaired fight-or-flight responses and heightened levels of fear and anxiety (9). Research has demonstrated that the suppression of “active” responses, such as fight or flight, may lead to “passive” reactions, also known as learned “passivity”. In this regard, activation of the PAG has been associated with “active” responses (54), particularly the dorsal part of the PAG (dPAG), which has been shown to participate in the activation of motor and autonomic components of active defensive responses (55). It has been shown that the PAG, the BNST and the amygdala receive significant serotonergic projections. Serotonin (5-hydroxitryptamine, 5HT) is mainly produced in the CNS by serotonergic neurons located in the dorsal RN (DRN) and the medial RN (MRN) (56–58). The DRN sends projections to the amygdala, particularly to the baso-lateral nucleus (BLA) and the CNA, the BNST, the PAG, some areas of the PFC, and to a lesser extent the ventral hippocampus (59–61) Activation of the DRN may lead to increased anticipatory anxiety, which plays an adaptive role in response to stressful situations (9, 32, 62), associated with increased arousal and alertness. Pre-clinical studies have shown that exposure to shock may lead to the immediate activation of the DRN, with the consequent release of 5-HT, in subjects exposed to both escapable or inescapable conditions (9). Activation of the DRN, with the consequent release of 5-HT in the amygdala, the BNST, and the PAG, has been associated with exposure to uncontrollable stressors (63, 64). Hence, in response to environmental stressors, the DRN receives excitatory input from the amygdala and other neural structures (65). It has been shown that the amygdala, particularly the CNA, sends projections to the DRN and to the locus coeruleus (LC) (66). Hence, in response to environmental stressors, the DRN receives excitatory input from the amygdala, primarily mediated by CRF, which stimulates the release of 5-HT in various neural structures (66, 67). Similarly, the LC receives excitatory input from the amygdala, also mediated by CRF, leading to the release of norepinephrine (NE) in different neural targets (66, 68), also associated with increased arousal and alertness. Noteworthy, the DRN receives noradrenergic projections from the LC (69), which exert a tonic excitatory control mediated by α1-adrenoceptors (70). The activity of serotonergic neurons in the DRN is self-regulated by inhibitory somato-dendritic 5-HT1A auto-receptors, which are activated by released 5-HT, thereby reducing further 5-HT release. However, high concentrations of 5-HT, resulting from intense DRN activation during inescapable stress, may down-regulate these auto-receptors, potentially neutralizing their normal inhibitory effect. This, in turn, may lead to increased 5-HT release by the DRN (71). Therefore, it has been proposed that inescapable stressful conditions may lead to the desensitization of 5-HT1A auto-receptors in the DRN, which could result in the sensitization of these serotonergic neurons, subsequently increasing 5-HT release (9). Hence, in response to uncontrollable stressors, serotonergic projections from the DRN to the PAG may result in the inhibition of active responses, while serotonergic projections from the DRN to the amygdala and BNST may provoke increased fear and anxiety.

Regarding neurobiological mechanisms involved in controllability, it has been shown that certain areas of the PFC, the hippocampus and the striatum participate in the assessment of perceived stressors and the detection of control. If stressors are perceived as controllable, activation of these neural structures may be translated into goal-directed behaviors, which differ from habitual behaviors (72). Specific areas in the ventro-medial PFC (VM-PFC), such as the pre-limbic PFC (PL-PFC), which has been proposed to be analogous to BA 32 in the human brain, participates in the acquisition of goal-directed associations (73), whereas the infra-limbic PFC (IL-PFC), which has been proposed to be analogous to BA 25 in the human brain, participates in habitual reward seeking (49). The PL-PFC sends projections to the dorso-medial striatum (DM-ST), which participates in the acquisition and expression of goal-directed actions (74), while the dorso-lateral striatum (DL-ST) participates in habitual reward seeking (75, 76). Hence, the PL-PFC and the DM-ST, which are involved in goal-directed associations, participate in control detection (9). The ventral striatum (V-ST), including the nucleus accumbens (NAc), plays a critical role in reward expectation, as well as goal-directed and motivated behaviors, also facilitating reward seeking (77, 78). It has been shown that activation of the VM-PFC may lead to inhibition of stress reactions, particularly mediated by activation of the amygdala and the DRN (79). Further research performed with humans showed that the VM-PFC expressed a differential response in controllable situations (39). Therefore, activation of the VM-PFC was associated with perceived control in humans and down regulation of stress responses, mediated by the activation of the amygdala and the DRN (39). The DRN receives projections from the VM-PFC, particularly from the IL and PL areas (80). Activation of these regulatory projections from the VM-PFC may lead to decreased activation of the DRN (81), as well as decreased activation of the amygdala (79), which in turn has been associated with decreased learned helplessness. In contrast, inhibition of the VM-PFC may increase symptoms of learned helplessness (81, 82). Hence, activation of the VM-PFC has been associated with perception of control and resilient behavior (79). It has been shown that the inhibitory effect exerted by the PL area of the VM-PFC on the DRN is mediated by glutamatergic projections, which exert stimulatory effect on inhibitory GABAergic interneurons (80). Hence, activation of these interneurons may be translated into an inhibitory effect on serotonergic neurons in the DRN. Likewise, glutamatergic projections from the VM-PFC may stimulate GABAergic interneurons in the amygdala, with the consequent inhibitory effect. Therefore, excessive activation of the amygdala may be counteracted by activation of the VM-PFC, hence decreasing feelings of fear and anxiety and improving subjective feelings of increased control. The amygdala also shares reciprocal connections with the DRN (83). It sends direct stimulatory projections from the CNA to the DRN mediated by CRF, or indirectly through projections from the BNST. The effects of CRF may vary depending on the specific receptor it binds to, whether it is released into serotonergic or GABAergic neurons, and the available ligand concentrations. Lower concentrations of CRF, associated with acute stressful situations, may bind mainly to CRF-R1, which has an inhibitory effect on 5-HT release, while higher concentrations, associated with chronic stressful conditions, may bind mainly to CRF-R2, which have a stimulatory effect on 5-HT release from the DRN (67). Hence, serotonergic projections from the DRN to the amygdala, particularly to the BLA, may lead to chronic anxiogenic states, which may be counteracted by the VM-PFC, which exerts inhibitory effects on the amygdala and the DRN.

The study of controllability in humans includes additional aspects, such as the ability to anticipate the impact of negative events, especially threatening situations, which are necessary for survival in an adverse environment. In this regard, the concept of predictability, which means the ability to predict the attainment of beneficial goals, as well as the capacity to avoid aversive outcomes, plays a critical role in the development of a sense of controllability. In human studies, the ability to anticipate future events may be highly adaptive. However, excessive anticipation of negative events may provoke feelings of anticipatory anxiety, which can be maladaptive (84–86). It has been proposed that anticipatory anxiety is linked to a subjective perception of anticipated lack of control over future events (39). In this regard, research on human subjects has also shown that the VM-PFC plays a crucial role in the subjective perception of control, particularly in anticipating aversive events (39). Activation of the VM-PFC has been also associated with decreased activity in the amygdala (87, 88), supporting the role of this topdown regulatory mechanism in humans (79). These findings reinforce the importance of these neural structures, as previously demonstrated in studies with non-human animals. Hence, in response to stressful situations, activation of the VM-PFC, with the subsequent inhibition of the amygdala and the DRN, is necessary to sustain the effect of subjective perception of control on emotional responses, which in turn may affect cognitive processing (39). Other areas of the PFC have been shown to participate in the regulation and activation of the VM-PFC. In this regard, the DL-PFC may induce activation of the VM-PFC (89, 90), which in turn may be translated into inhibition of the DRN and the amygdala. The DL-PFC plays a critical role in cognitive functions, such as working memory (91, 92, and decision making (93), while the VM-PFC has been associated with emotional functions, such as emotional regulation (94, 95), and it has been shown to participate in value-based decision making (96). These interactions between both areas in the PFC highlight the importance of the interplay between cognitive processing and emotional processing, particularly in stressful situations that require rapid and effective implementation of adaptive responses. The DL PFC is also reciprocally connected with the dorsomedial PFC (DM-PFC) and the ventrolateral PFC (VL-PFC), forming a circuit involved in the regulation of the amygdala, which in turn may be further translated into emotion regulation (97).

## Therapeutic strategies and future developments

Learned helplessness has been associated with alterations of the serotonergic system, particularly increased activation of the DRN, associated with passive behavioral responses, and decreased activation of the MRN, associated with decreased tolerance over adverse events. Conversely, the perception of control over adverse events has been associated with increased activation of the VMPFC, the DLPFC, and the MRN. It has been shown that the VMPFC contributes to emotional regulation and the inhibition of subcortical structures, such as the amygdala and the DRN, while the DLPFC supports goal-directed behavior and instrumental learning. Both areas are also innervated by serotonergic projections. Hence, under conditions of adequate serotonergic tone, their function is enhanced, allowing for improved evaluation of action and the restoration of perceived control. In contrast, dysregulation of these circuits may underlie the generalized passivity observed in states of learned helplessness.

Numerous studies have demonstrated that antidepressants, particularly selective serotonin reuptake inhibitors (SSRIs), can effectively reverse passive behavioral responses induced by uncontrollable stress in animals, hence recovering the ability to elicit active responses. The therapeutic effects of SSRIs involve the modulation of multiple mechanisms associated with certain neural structures and specific receptors, mainly mediated by different 5-HT receptor subtypes. In the amygdala, SSRIs downregulate excitatory 5-HT2A receptors and enhance the function of inhibitory post-synaptic 5-HT1A receptors, leading to reduced emotional reactivity and attenuation of fear responses (98, Hensler et al, 2006, 99). This receptor-level modulation contributes to diminished limbic hyper-reactivity and facilitates prefrontal top-down regulation. In the hippocampus, SSRIs promote BDNF-dependent neuroplasticity and neurogenesis primarily through activation of post-synaptic 5-HT1A receptors, supporting contextual processing of stress and emotional memory integration (82, 100–102). Within the VMPFC, increased serotonergic neurotransmission, mediated mainly by post-synaptic 5-HT1A receptors, contributes to the suppression of subcortical stress responses by enhancing prefrontal inhibition over the amygdala and the DRN, thereby reducing passive, generalized stress responses and enabling context-sensitive appraisal of controllability (9, 103–105). This mechanism is critical to the transition from more reflexive, emotion-driven responses, to more cognitive-driven responses, including goal-directed coping behavior. In the DLPFC, SSRIs modulate executive function and goal-directed behavior through binding to post-synaptic 5-HT1A and 5-HT2A receptors, improving executive control, cognitive flexibility and instrumental learning, which are necessary to detect and consolidate action-outcome contingencies (98, 104, 106). Together, these receptor-specific effects promote a neurobiological setting in which adaptive coping strategies and the perception of control may be recovered and strengthened, which in turn may lead to recover the capacity to distinguish between controllable and uncontrollable stressors, thereby reversing learned helplessness and promoting learned controllability. (9, 82, 98, 103).

Although the effects of SSRIs have been widely studied in relation to mood and anxiety, further research is needed to examine their potential role in restoring the perception of control. Investigating their effects within paradigms that explicitly assess controllability could offer new insights into the biological mechanisms underlying recovery from learned helplessness and the development of learned controllability.

In addition to psychopharmacological approaches, future research should further investigate the potential of neuromodulatory interventions, such as transcranial magnetic stimulation (TMS), to enhance perceived control. Considering the central role of prefrontal regions in instrumental learning, goal-directed behavior, and emotion regulation, TMS may serve as a valuable tool to strengthen the neural circuits underlying the subjective experience of control. This effect may be particularly effective when TMS is combined with behavioral tasks specifically designed to reinforce the sense of agency and facilitate the learning of action–outcome contingencies. Such integrated neurocognitive approaches could open new avenues for mechanism-based treatments targeting disorders characterized by learned helplessness, maladaptive coping, and impaired stress resilience.

Regarding psychotherapeutic strategies, cognitive therapy contributes to restoring the perception of control by restructuring maladaptive beliefs related to self-efficacy and contingency. By modifying cognitive appraisals of stressors, such interventions engage prefrontal regions, such as the DL-PFC, involved in top-down regulation, thereby attenuating limbic overactivation. Moreover, therapeutic paradigms based on experiential re-exposure to controllable stressors can reactivate instrumental learning circuits and strengthen the encoding of action-outcome contingencies. These experiences facilitate the development of adaptive coping strategies and may serve to reverse helplessness by enhancing functional connectivity between the DL-PFC, the VM-PFC, the striatum, and limbic areas, such as the amygdala. Thus, therapies that restore the subjective sense of control may have significant neurobiological and clinical impact in the treatment of stress-related disorders.

More recently, we have initiated the development of therapeutic strategies based on virtual reality (VR). Given the central role of perceived control in modulating stress responses and promoting adaptive coping mechanisms, VR represents a promising avenue for intervention. VR environments enable the creation of immersive and interactive scenarios in which individuals can actively engage in goal-directed behaviors and experience clear cause–effect relationships between their actions and outcomes, thereby facilitating the development and consolidation of a subjective sense of controllability. Moreover, the adaptability of VR-based interventions to individual needs supports the implementation of personalized therapeutic strategies that not only aim to reverse learned helplessness, but also foster long-term psychological resources to enhance perceived control, resilience, and emotional well-being.

## Conclusions

It has been shown that prolonged and sustained exposure to uncontrollable stressors may lead to learned helplessness, i.e. inability to escape or avoid aversive situations even when escape/avoidance are possible, also interpreted as a perception of lack of control. In “learned helplessness” an organism learns that outcomes are independent of their actions, and it was suggested that in humans this process involves, in addition to a subjective perception of helplessness, a learned attitude of decreased motivation to exert any effort or coping with perceived stressors.

Conversely the sense of control offers the ability to effectively respond to environmental stressors by taking purposeful actions through goal-oriented behaviors. This ability, which may be stimulated, trained or learned, constitutes a process referred to as “learned controllability”. Learned controllability thus might play a role in the development of both psychological and neurobiological aspects of resilience. (11, 79). If so, the ability to perceive control may have a regulatory effect on emotional reactions triggered by adverse situations, whereby a subjective perception of control plays a crucial role in developing more effective coping strategies and achieving more successful behavioral outcomes. This may lead to improved decision-making and goal-directed actions, ultimately reinforcing the subjective perception of controllability.

Neurobiologically learned helplessness had been linked to increased activity in the DRN and decreased activity in the MRN, as well as hyperactivation of the amygdala. These changes are accompanied in the long run by alterations in the HPA axis and reduced activation of the VMPFC (9, 107, 108). Heightened perception of control, on the other hand, was linked to increased activation of the MRN, with the consequent regulatory effect on the ventral hippocampus and, subsequently, the NAc. In humans it has been associated with increased activation of the DL-PFC and the VM-PFC, which may play a role in decreasing activation in the amygdala and the DRN. These changes have been associated with improved tolerance to adversity, decreased anxiety, and the development of resilience.

Controllability, similarly to learned helplessness, is associated with instrumental learning, where an individual learns to associate a voluntary action with a desired result (11), where associations between behavioral responses and effective outcomes, are learned. Behavioral responses are directed toward achieving desired outcomes, aimed at effectively coping with perceived stressors, such as avoiding aversive situations or approaching favorable stimuli. Establishing a causal link between voluntary actions and desired outcomes enhances subjective perception of control, which may be perceived as rewarding, hence providing positive feedback to the learning process. In this regard, it has been observed that striatal activity can be influenced by the perceived relationship between a voluntary action and its outcome (109). Moreover, the emotional experience of perceiving control, manifested through decision-making behavior, has been associated with activity in cortico-striatal systems involved in reward processing (110). Hence, the perceived efficacy of voluntary actions in achieving desired goals provides essential feedback for developing a subjective sense of controllability. On a cognitive level, the goal-directed system assesses potential changes in outcomes, enabling the modification of behavioral strategies as needed (111). It has been demonstrated that the goal-directed system also participates in the detection of control over adverse situations, which plays a critical role in the development of more effective coping strategies and therapeutic resources. In this regard, it has been shown that previous experiences of control over adverse situations may reduce the susceptibility to helplessness, hence providing “immunization” against certain stressful conditions (9, 112), which then may be recognized as “controllable”. This is important because the therapeutic focus stemming from learned helplessness has been on reversing the harmful impacts caused by a sense of uncontrollability (11). It has been shown that animals initially exposed to escapable shocks may learn to react with an active response and not become passive, even when faced with inescapable shocks later in life (113). Since “controllability” in humans may be understood as feeling that emerges from instrumental learning and higher-level cognitive processing, it is possible to conceive therapeutic strategies based on this learning process. Hence, individuals exposed to chronic stressful situations may be able to develop a subjective sense of control, which may be translated into more adaptive responses rather than the development of learned helplessness. In addition, it could be possible to conceive the development of preventive strategies, aimed at teaching and training vulnerable individuals, mostly during early periods of life, to develop a subjective feeling of control.

Further delineating the psychological and neurobiological mechanisms underlying cognitive and emotional processes involved in learned helplessness and learned controllability might be an important step for advancing modern psychiatry. Therefore, ongoing research and further advances in understanding these mechanisms may lead to the development of more effective biological and therapeutic approaches for the prevention and treatment of depression, as well as more efficient strategies for fostering resilience.

## Literature search approach

The literature reviewed in this manuscript was selected to support the central hypothesis that learned helplessness and learned controllability represent opposing processes with implications for therapeutic intervention. Rather than following a systematic review protocol, we adopted a targeted narrative approach, identifying relevant publications across a broad temporal range through searches in PubMed, Scopus, and Web of Science. Search terms included combinations of learned helplessness, learned controllability, perceived control, serotonin, stress, prefrontal cortex, amygdala, and antidepressants. Additional sources were identified via manual screening of reference lists from key conceptual and empirical articles. Priority was given to studies that informed the neurobiological and therapeutic dimensions of the argument.
